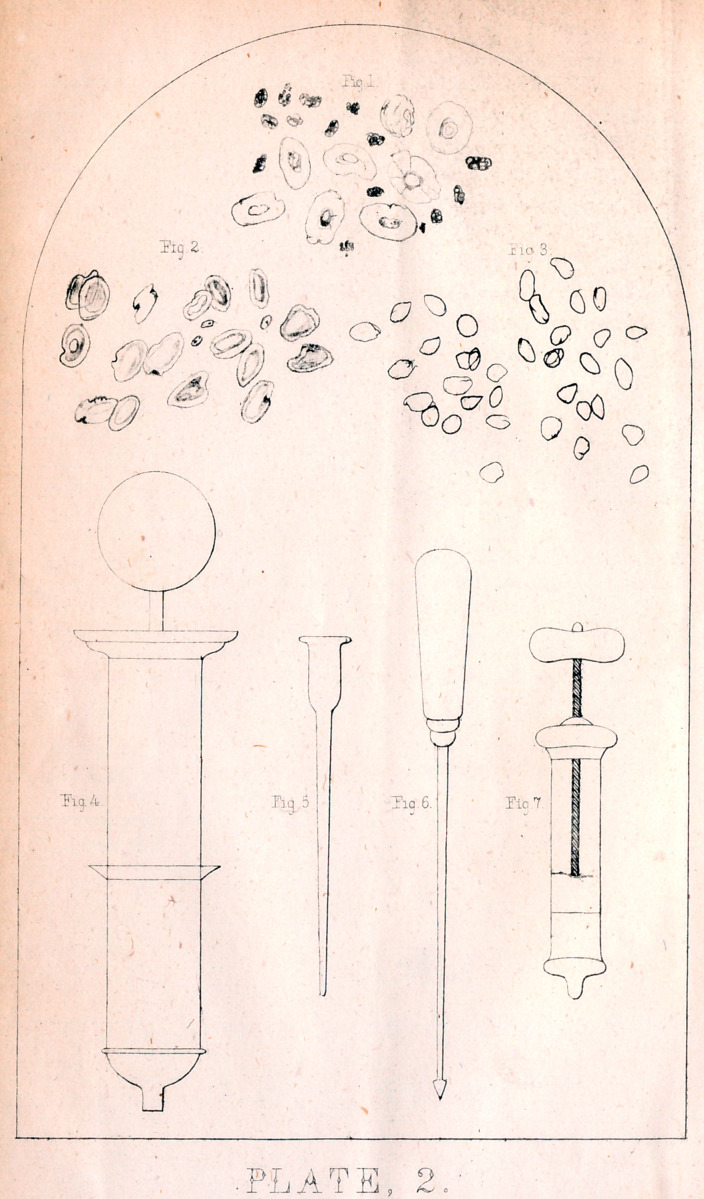# Indiana Medical Journal

**Published:** 1854-07

**Authors:** 


*Indiana Medical Journal.*

We have received the first number of this new auxiliary to our periodical literature. We predict for it a successful career, should future numbers equal in merit that already received.

There are two difficulties to be met in conducting such an enterprise. The first relates to its *pecuniary* support. We are satisfied from our experience in Journalism, that the only safe course is to require payment invariably in advance, and to stop sending the Journal at the expiration of the time paid for. This course enables the publisher always to know with certainty the amount of income, and saves him the disappointment always resulting from estimates based on a large list of non-paying subscribers. It is an accommodation to subscribers themselves. Of this, we are quite certain. It is much easier to pay two or three dollars and feel no more uneasiness or anxiety on the subject, than to pay eight or ten after two or there years of torture under the lashes of a guilty conscience, or, if there is no conscience in the matter—as we sometimes fear—the vexation of continual duns.

Prepayment secures a better Journal. Editors and Publishe do their work more cheerfully and more thoroughly after receiving from their readers the only unmistakeable evidences of appreciation.

The second difficulty to be met in sustaining a Medical Journal relates to its matter. It is true that two or three men can write enough during a month to fill the original department of a Journal, but they cannot give a sufficient variety of matter to interest and instruct all classes of readers. In order that this may be done each member of the profession should recognise a personal responsibility resting upon him, he should remember that while he receives his two, three, or more Journals, as the case may be, and pays for them in advance, he is only paying for the mechanical part of them, while for the matter of which they are made up and which, alone, constitutes their intrinsic value, he is indebted to the profession at large. This indebtedness can only be repaid by contributing in turn something from which others shall derive profit. We have no right to use the weapons of our art without assisting in their multiplication and improvement; we are not at liberty to enter the field of our science except it be to cultivate and develop from its fertile soil new truths.

We have not written the above with reference to the *Indiana Medical Journal*. We are glad to see that it is well filled with articles from the able pens of our brethren in Southern Indiana. We hope this will continue to be the case, but, in regard to a majority of our Medical Periodicals, it is too true that they do not find that support—by way of contributions—from their readers that their Editors have a right to expect.

We are writing now for each and every one of our own readers, and we say to them, would you have the mechanical part of the Journal properly and neatly executed.—let every one who has not done so already, send us the *money* Would you have its pages filled with useful and interesting matter, select from the varied store of facts gathered in the discharge of your daily duties an offering to strengthen the hands and encourage the hearts of your brethren. J.

### EXPLANATION OF PLATE I.

*All the figures in this plate are magnified six hundred and twenty diameters.*

FIGURE 1. Natural appearance of the blood corpuscles of a healthy pigeon Some are seen edgewise.

FIG. 2. Blood treated with a solution of iodine 10 grs., potass iodide 30 grs., aquæ, 13. The globules are nearly natural, though a little more round, and the color is brought out.

FIG. 3. Blood on which a little infusion woorara was dropped, and at the same time some of the solution of iodine and iodide potass. Some of the globules appear preserved, though they are very much reduced in quantity, the debris of the corpuscles being the small spots, appearing like nuclei.

FIG. 4. Blood treated with a solution of woorara.

FIG. 5. Blood taken half an hour after death, from the pectoral muscle of a pigeon which had been injected with eight drops of a solution of woorara—taken from the side opposite to that injected.

FIG. 6. Blood taken from the heart of a pigeon which died in five minutes after woorara was injected. The corpuscles have all lost their natural shape, and have a tendency to run into one another.

FIG. 7. Blood taken from the medulla oblongata of a pigeon two hours after he died from an injection of eighteen drops of a solution of woorara.

Fig.1.

Fig 2.

Fig. 3.

Fig.4.

Fig. 5

Fig. 6

Fig. 7.

PLATE, 1.

PLATE, 2.

### EXPLANATION OF PLATE II.

*Magnified six hundred and twenty diameters.*

FIGURE 1. Frog's blood treated with solution of woorara. Globules not all taken from one field as they were broken down.

FIG. 2. Blood from head of pigeon dead from bite of rattlesnake, taken fifteen hours after death

FIG. 3. Pigeons' blood to which the venom of a rattlesnake had been added

FIG. 4. Syringe for infiltration—natural size.

FIG. 5. Syringe for measuring the poison by drops—natural size.

FIG. 6. Canula of trochar to which the two syringes are adapted—natural size.

FIG. 7. Stylet of trochar—natural size.

## Figures and Tables

**PLATE, 1. f1:**
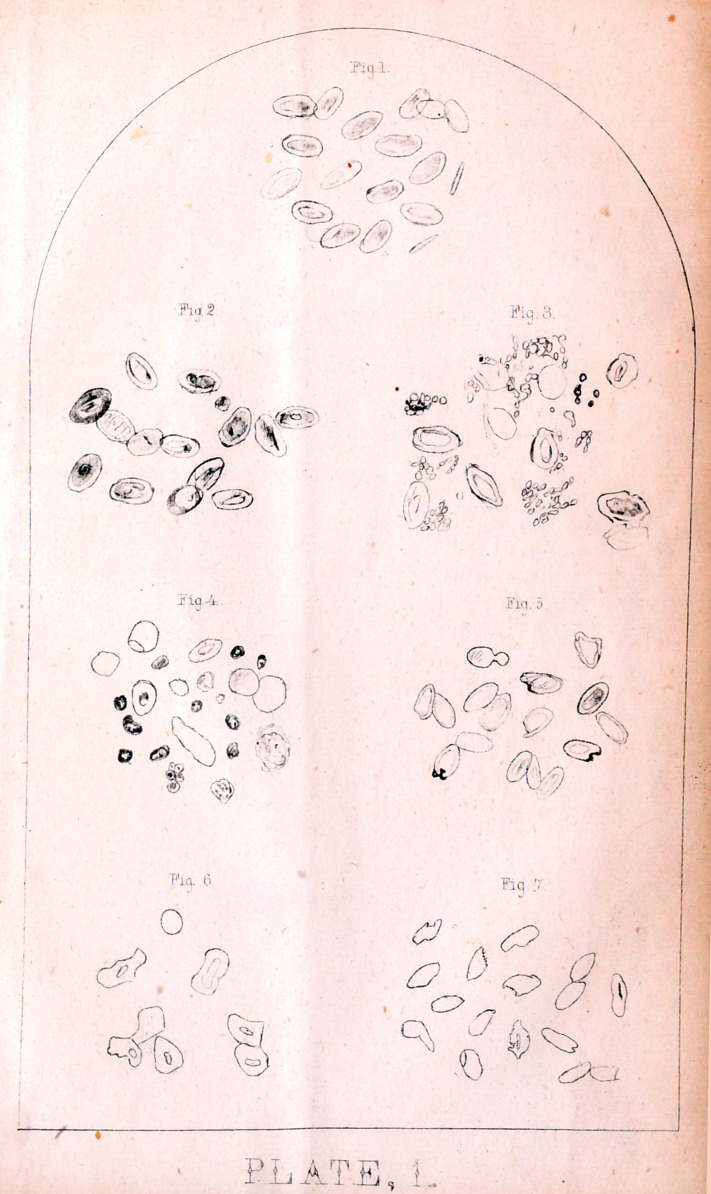


**PLATE, 2. f2:**